# Development and validation of burnout factors questionnaire in the operating room nurses

**DOI:** 10.1038/s41598-024-56272-2

**Published:** 2024-04-08

**Authors:** Esmaeil Teymoori, Armin Fereidouni, Mohammadreza Zarei, Saeed Babajani-Vafsi, Armin Zareiyan

**Affiliations:** 1 Department of Operating Room Technology, School of Paramedical Sciences, Aja University of Medical Sciences, Tehran, Iran; 2 Department of Operating room technology, Community Based Psychiatric Care Research Center, School of Nursing and Midwifery, Shiraz University of Medical Sciences, Shiraz, Iran; 3 Autoimmune Diseases Research Center, Kashan University of Medical Sciences, Kashan, Iran; 4 Public Health Department, Health in Disaster & Emergencies Depatment, Aja University of Medical Sciences, Tehran, Iran

**Keywords:** Psychometrics, Validation, Burnout, Operating room, Nurses, Psychology, Health occupations

## Abstract

Nurses may experience burnout more than other professions. Occupational burnout is a serious concern considering the importance of nurses' jobs in patient care. This study was carried out with the aim of designing and validating the questionnaire of burnout factors in the operating room nurses. Mixed method study was done in two qualitative and quantitative phases in 2022 on Iranian operating room nurses. In the first stage, the concept of operating room nurses' burnout was explained using interviews and literature review, and items were generated. In the second stage, the face validity, content and construct validity of the questionnaire was examined with 342 operating room nurses, and also the reliability of the questionnaire was tested using internal consistency (Cronbach's alpha) and stability (test–retest). After conducting the interview and literature review, 65 questions were extracted. Based on face validity, 4 items were modified. After content validity, 40 items remained. In construct validity, after exploratory factor analysis, 34 items with 5 dimensions were extracted. These dimensions included Organizational, Individual, Interpersonal, Occupational Nature and Managerial factors. Cronbach's alpha and intra-class correlation coefficient were equal to 0.937 and 0.946, respectively. The designed tool based on understanding the concept of burnout in operating room nurses has appropriate and acceptable validity and reliability. Therefore, it can be used to measure burnout in operating room nurses.

## Introduction

For the first time, burnout was defined by Freudenberger in 1974 with a state of internal fatigue in people who have the task of helping others^1^. Most nurses have experienced burnout syndrome. This syndrome may be accompanied by physical, emotional and mental fatigue and is shown by symptoms of depersonalization and reduced interest in work^2–4^.

Studies have shown that nurses may experience burnout more than other professions. Occupational burnout is a serious concern considering the importance of nurses' jobs in patient care^5,6^. Burnout can lead to mental health diseases such as hopelessness, depression and suicide of personnel^7^. Also, burnout can affect individual and professional communication and cause personnel to leave the profession^8^. The most recent study by the American Medical Association reported that two out of five nurses intend to leave their current practice^9^.

The operating room environment has unique characteristics for nurses. Operating room nurses need to work long hours in stressful surgeries with high concentration^10^. Also, operating room nurses are highly exposed to biological, chemical and physical risks. These risks include constant exposure to disinfectants, X-rays, sharp items, anesthetic gases, physical injuries caused by standing for long periods and holding surgical instruments. All these cases can lead to increased burnout in operating room nurses^11^. These negative effects may increase job pressure and reduce the quality of patient care and ultimately patient safety^12^. An extensive study in the United States in which more than 7000 health care professionals participated showed that there is a significant correlation between job burnout, medical error, and patient safety, so that reducing job burnout can improve patient safety^13^. Investigating and coping with burnout in the operating room may be a priority not only to support surgical team members, but also to improve the safety and quality of patient care^14^.

Burnout is considered a long-term response to chronic workplace stress^15^. The risk of burnout in an environment with high job stress is seven times more than in an environment with low job stress^16^. Operating room nurses endure high stress both physically and mentally^17^. This long-term stress reduces resilience and increases burnout of operating room nurses. Nurses who work in the operating room for a long time may have a negative attitude towards their workplace and become extremely nervous, so it is necessary to investigate burnout in this particular group^18^. Several factors such as anxiety, lack of empathy, high workload, work environment culture and lack of personnel support by managers may cause burnout^9,19^. Therefore, considering various factors in burnout, there is a need to design valid and reliable tools to measure it in operating room nurses. Several tools have been designed in the field of burnout, such as Jones Burnout scale^20^, Pines Burnout measure^21^, Geldard burnout inventory^22^, Shirom-melamed burnout Measure^23^, Oldenburg Burnout Inventory, Copenhagen Burnout Inventory and Maslach Burnout inventory^24^. The Maslach Burnout inventory has been used in Iran and many countries, but the tools are general and non-specific and have low sensitivity for measuring in health environments^25^. Since burnout can affect the performance of nurses in the operating room and ultimately affect the patient's safety, also considering the need to provide accurate and suitable tools with the work environment in order to determine the factors of burnout, therefore the present research was carried out with the aim of designing and validating the questionnaire of burnout factors in operating room nurses in Iran.

## Methods

### Aim and research questions

Design and validation of burnout factors questionnaire in operating room nurses.

Does the occupational burnout questionnaire of operating room nurses have face and content validity?

Has the construct validity of the occupational burnout questionnaire of operating room nurses been measured?

How is the reliability of the burnout questionnaire of operating room nurses determined?

### Study design

**Figure 1:**
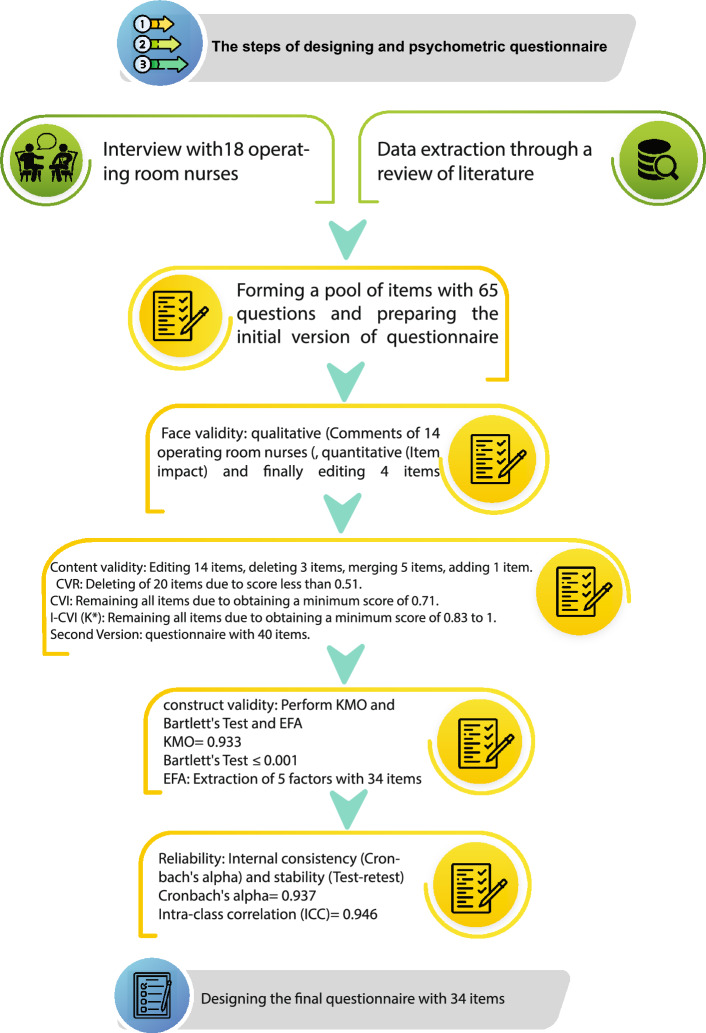
Procedures for designing the burnout factors questionnair of operating room nurses.

The present study is a mixed method research that was conducted in two qualitative and quantitative stages (Item generation, questionnaire design, item reduction and instrument validation) in 2022 on Iranian operating room nurses. The study was conducted in the 7 hospitals of Iran in Tehran city (Imam Reza, Besat, Imam Khomeini,Firoozgar, Talaghani, Baqiyatallah, and Rasoul Akram). All methods were performed according to the relevant guidelines and regulations.Qualitative stage is designed based on COREQ (Consolidated criteria for reporting qualitative research) guideline^26^. Quantitative stage is designed based on COSMIN (consensus-based standards for the selection of health status measurement instruments) guideline^27^. Procedures for designing the burnout factors questionnaire of operating room nurses are shown in Fig. 1.

### Sample

In the qualitative phase of the study, 18 operating room nurses were interviewed until the data saturation phase. In the quantitative phase of the study, 14 nurses from the operating room were included in the study. In the content validity part, 14 operating room specialists and experts in instrument design, psychology and nursing were polled. In the construct validity part, 342 operating room nurses were asked to complete the burnout questionnaire. Also, for the reliability of the questionnaire, 342 operating room personnel were used to determine Cronbach's alpha and 30 people were used to determine test–retest. Sampling method was convenience in this study. In the following, the sampling details are explained in each step.

### Stage 1: questionnaire development

This stage was done with the qualitative approach of conventional content analysis. For the interview, the first participant was included in the study based on the inclusion criteria, Including the ability to communicate properly and high work experience in the operating room, at least an associate's degree in operating room nursing, willingness to participate in the study and not suffering from history of psychological diseases and not taking drugs related to psychological diseases according to the participants' self-report.Exclusion criteria were failure to complete the interview process, and transfer to another hospital or retirement.

Next, other participants were included in the study with purposeful sampling and maximum variation until data saturation. Finally, an individual, face-to-face, in-depth and semi-structured interview was conducted with 18 operating room nurses. The duration of the interview was between 40 and 80 min on average, and sometimes some participants were re-interviewed to complete the information. The interview environment was in the rest room of the personnel or the supervisor's room and in a calm condition. The interview started with a general question such as: “Describe your experience of a stressful day in the operating room” and with probing questions such as: “Explain more about this?” or “Can you give an example?” followed based on previous answers and the purpose of the study. All interviews were fully recorded and typed in word software and entered into MAXQDA-2020 software. Then, the data analysis of the interviews was carried out using the steps suggested by Graneheim and Lundman^28^. In this method, the content of each interview was recorded and typed verbatim, and a general understanding was obtained after several readings. Then the text was divided into meaning units and a code or codes were assigned to each unit. The codes were classified into classes based on similarities and differences and were used to produce items. Then, the number of items was completed with literature review. In the present study, four criteria of credibility, dependability, confirmability, and transferability, which were presented by Guba & Linklen, were used to ensure the accuracy and validity of the data^29^. The item development steps are briefly shown in Table 1.

**Table 1:** Briefly description of the steps of item development.

Item development		
Step 1	Individual, face-to-face, in-depth and semi-structured interview with 18 operating room nurses based on inclusion criteria to data saturation	**Interview time:** 40–80 min **Interview environment:** rest room of the personnel
Step 2	All interviews were fully recorded, typed and coded	Type in word software 2019 and coded by MAXQDA- software 2020
Step 3	Determining meaning units from the text of the interviews Classification of codes based on similarities and differences and completed with literature review	**For ensure of the trustworthiness and reliability of the data used:** four criteria (credibility, dependability, confirmability, and transferability)
Step 4	Classification of interview answers based on codes and production of initial questionnaire items	Based on the comments of experts included nursing professor, surgeon, psychologists and expert

### Stage 2: validity and reliability

In order to prepare the primary tool, the questions resulting from the qualitative content analysis and literature review based on the comments of the research team, experts in the operating room, experts in the field of psychology and psychometrics, similar and repetitive items were removed or merged. Also, some phrases or words were re-surveyed. After the changes made, the initial questionnaire with 65 statements was prepared for the psychometric process.

### Face validity

In order to check face validity, in the qualitative part of the questionnaire items were checked in terms of difficulty, irrelevancy and ambiguity. In this regard, 14 operating room nurses were asked to provide their comments after reviewing the questions to be used in the subsequent analysis. The items suggested were reviewed and corrected. Next, Item impact method was used to calculate the face validity of the questionnaire in a quantitative method. The importance of each item in a 5-point Likert scale including completely important (5 points), somewhat important (4 points), moderately important (3 points), slightly important (2 points) and not important (1 point) was investigated. In this part, if the impact score of each item was higher than 1.5, it was recognized as suitable for further analysis and was retained^30^.

### Content validity

Content validity was checked with two qualitative and quantitative methods. In the qualitative part, questionnaires were given to 14 operating room specialists and specializing in instrument design, psychology and nursing. Expert comments about grammar, wording, item allocation and scaling were reviewed. In order to check content validity quantitatively, Content Validity Ratio (CVR) and Content Validity Index (CVI) were used. First, to determine CVR, 14 experts (8 nursing professor, 2 surgeon, 3 psychologists and 1 expert in psychometrics of the questionnaire) were asked to express their comments about the necessity of each item in a 3-point Likert scale (necessary—useful but not necessary and not necessary). Thus, at this stage, based on Lawshe's table, items with CVR less than 0.51 (based on the evaluation of 14 experts) were removed^31^. To determine the CVI, it was done based on the content validity index of Waltz and Basel^32^. For this purpose, the researchers provided the designed questionnaire to 12 experts (7 nursing professor, 1 surgeon, 3 psychologists and 1 expert in psychometrics of the questionnaire)) and asked them to determine the three criteria of relevance, Simplicity, and Clarity of each of the questionnaire phrase in a four-part Likert scale. CVI was calculated for items that scored 3 and 4 using the following formula^33^. According to the Lynn table, the condition for accepting the item when the number of experts is 12 is 0.79^34^.

$$CVI = \frac{{{\text{Number of raters giving a rating of }}3{\text{ or }}4}}{{\text{Total number of raters}}}$$

Cohen's kappa coefficient (K*) was calculated using Polit et al.'s method and using the following formula. Based on the obtained score, kappa evaluation was done (less than 0.6 = poor, 0.6 to 0.74 = good, and above 0.74 = excellent)^35^.

$$pc = \left[ {\frac{{\left[ {N!} \right]}}{{\left[ {A!\left( {N - A} \right)!} \right]}}} \right] \times 0.5^{N}$$

$$K = \frac{{{\text{I}} - {\text{CVI}} - pc}}{{1 - {\text{pc}}}}$$

K = Modified Kappa coefficient.

Pc = Probability of random correlation coefficient.

N = Number of experts.

A = Number of very important scores (3 or 4).

### Construct validity

In the present study, factor analysis was used to determine the construct validity. Before doing the factor analysis, the initial internal consistency was done on 30 operating room nurses. It is also necessary to consider the adequacy of the sampling before the factor analysis. Sampling adequacy means whether the number of available data is suitable for factor analysis or not. For this purpose, the Kaiser–Meyer–Olkin (KMO) index and Bartlett's Test can be used. The score of KMO more than 0.7 is appropriate^36^. Also, the significance level of Bartlett's Test is less than 0.05 acceptable^37^. Exploratory Factor Analysis (EFA) was used as the most common method to determine construct validity. Principal component analysis (PCA) method was used to extract the factors and PROMAX rotation was used for the interpretability of the factors^38^. The minimum acceptable factor loading was considered to be 0.3^39^. The minimum sample required for exploratory factor analysis is three to ten participants per item^36^. In the present study, 370 questionnaires were distributed among operating room nurses and finally 342 questionnaires were completed and collected by the samples. Inclusion criteria included at least an associate's degree in operating room and more than one year of work experience in the operating room, willingness to participate in the study and not suffering from history of psychological diseases and not taking drugs related to psychological diseases according to the participants' self-report. Exclusion criteria were failure to complete the questionnaire, and transfer to another hospital or retirement.

### Reliability

To perform reliability, two methods of internal consistency and stability of the questionnaire were used. To calculate internal consistency, Cronbach's alpha coefficient was used with a sample size of 342 personnel. Also, for stability, test–retest method was used with a sample size of 30 people and with a time interval of two weeks^40^. Cronbach's alpha acceptable for the questionnaire between 0.7 to 0.8 is^41^. The most acceptable test to calculate the level of stability is the intraclass correlation coefficient (ICC) test, if this index is higher than 0.80, the level of stability is acceptable^42^. Thus, at this stage, based on Lawshe's table, items with CVR less than 0.51 (based on the evaluation of 14 experts) were removed^31^.

### Data analysis

For demographic information, descriptive analyzes including frequency and percentage were used.

For the validity of the questionnaire, face (Impact score), content (CVR and CVI) and construct validity (KMO, bartlett’s test of sphericity, a scree plot, principal component analysis and promax rotation) were used. The reliability of the questionnaire was measured using the two tests of internal consistency (Cronbach’s alpha coefficient) and stability (test–retest).

For face validity if the impact score of each item was higher than 1.5, it was recognized as suitable for further analysis and was retained^30^. For CVR, based on Lawshe's table, items with CVR less than 0.51 (based on the evaluation of 14 experts) were removed^31^. For CVI based on the Lynn table, the condition for accepting the item when the number of experts is 12 is 0.79^34^. For construct validity the score of KMO more than 0.7 is appropriate^36^. Also, the significance level of Bartlett's Test is less than 0.05 acceptable^37^. The minimum acceptable factor loading was considered to be 0.3^39^. Cronbach's alpha acceptable for the questionnaire between 0.7 to 0.8 is^41^. The most acceptable test to calculate the level of stability is the intraclass correlation coefficient (ICC) test, if this index is higher than 0.80, the level of stability is acceptable^42^.SPSS software version 2022 and MAXQDA software version 2020 were used for data analysis.

### Ethical considerations

The permission of this research was approved by the ethics committee of AJA University of Medical Sciences with ethics code IR.AJAUMS.REC.1399.277 and access link https://b2n.ir/n92193. At the beginning of the study, the research objectives were explained to the participants and written informed consent was obtained. The participants were assured about maintaining the confidentiality of the research data. In addition, it was explained to them that they have the right to withdraw from the study at any stage of the research.

## Results

The concept of burnout was determined after qualitative content analysis of interviews with 18 operating room nurses. The characteristics of the interview participants are shown in Table 2. According to operating room personnel, burnout is a mental concept that is influenced by Organizational, Individual, Interpersonal, Occupational Nature and Managerial factors. Then, in addition to the interviews, a literature review was also conducted and the items of the questionnaire were extracted. Finally, after removing, integrating and re-surveyed similar and repetitive items and using the comments of experts, a pool of questions with 65 items was designed and prepared for psychometrics. The Likert scale of the questions was designed as 5 parts including “never, rarely, sometimes, often, always”. The lowest score for each statement is zero due to choosing the “never” option and the highest score is four due to choosing the “always” option.

**Table 2:** Demographic characteristics (N = 18).

Variables	Category	N	Percent
Gender	Female	10	44.4
	Male	8	55.6
Marital status	Single	6	33.3
	Married	12	66.7
Educational degree	Associate degree	2	11.1
	Bachelor’s	11	61.6
	Master’s	5	27.8
Shift	Fixed	8	44.4
	Variable	10	55.6
Employment status	Permanent	15	83.3
	Contractual	3	16.7
Working in another hospital	Yes	9	50
	No	9	50
Managerial position	Yes	7	38.9
	No	11	61.1

Face validity: In the qualitative stage of face validity, items No. 4, 5, 37 and 51 were modified in terms of writing. And in the face validity quantitative section, all the items were retained due to the Item Impact score higher than 1.5.

Content validity: at this stage, items number 4, 6, 10, 11, 12, 14, 16, 17, 28, 30, 32, 33, 35, 53, edited, items 7, 20, 21, and items 42, 43 And 44 was merged and item 55 was merged with 56. Finally, one item was added to the collection of items. After this stage, the number of questions in the questionnaire reached 60 questions. After calculating the CVR, according to the experts' comments, a decision was made to retain or remove the item. Thus, at this stage, items with CVR less than 0.51 were removed. Based on this, 20 items were removed from the 60-item questionnaire. After calculating the CVI, considering that the scores of the items were higher than 0.79, no item was removed and a questionnaire of 40 questions was obtained. The value of Scale-CVI/Ave was also equal to 0.97. The I-CVI (K*) of the whole item was obtained from 0.83 to 1, so all the items were at an excellent level and were retained.

**Figure 2:**
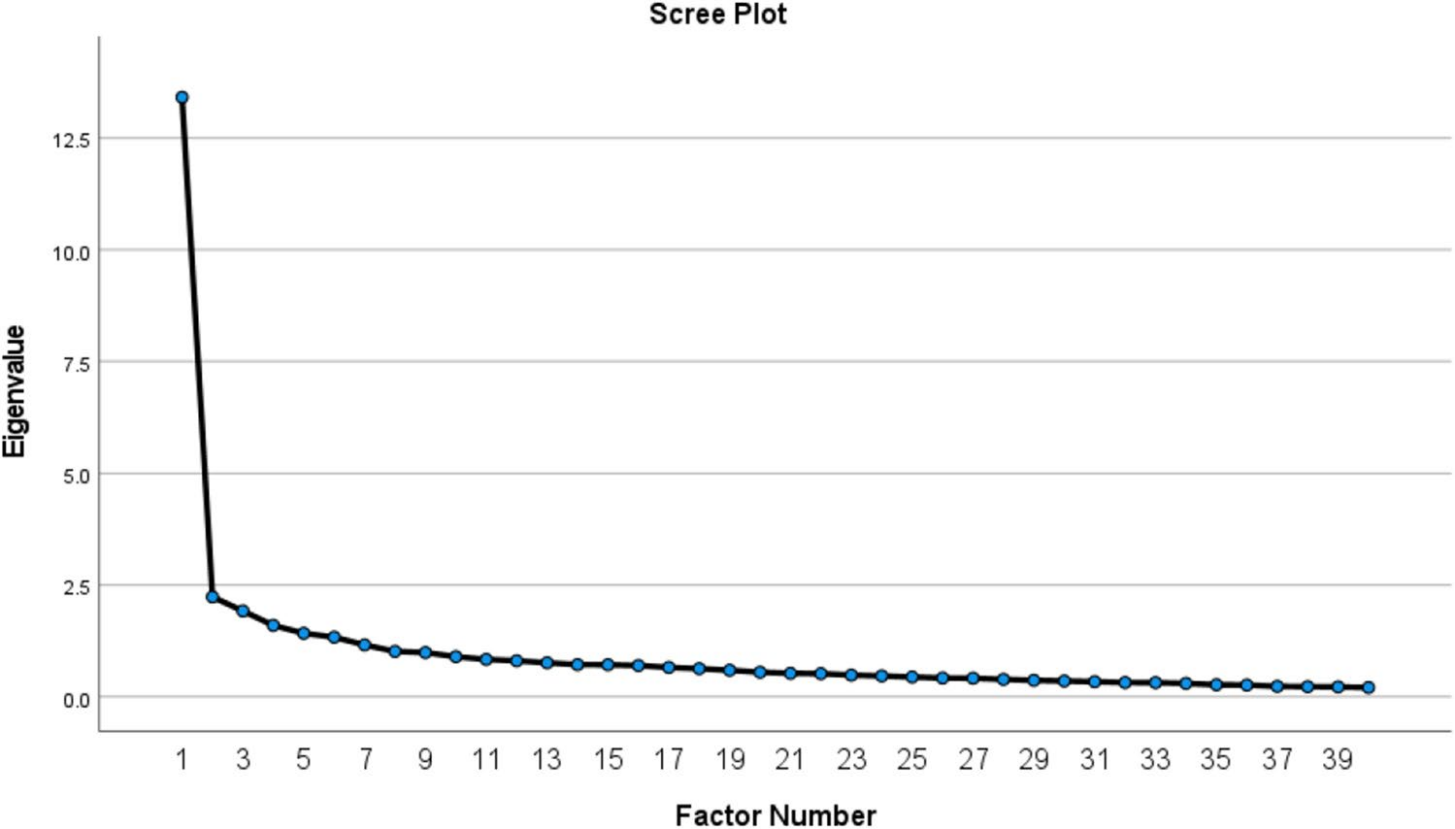
Scree plot to determine the number of factors in the questionnaire of burnout factors in operating room nurses.

The initial internal consistency of the questionnaire was obtained using Cronbach's alpha coefficient equal to 0.93. The value of KMO and Bartlett's Test is specified in Table 3. In PCA implementation of the 40-item questionnaire, factor coefficients greater than 0.3 were considered as factor loadings. After performing EFA and Promax rotation, according to Eigenvalue above one, KMO index and Scree Plot (Fig. 2), five factors (Organizational, Individual, Interpersonal, Occupational Nature, Managerial) with 34 items were extracted (Table 4).

**Table 3:** Kaiser–Meyer–Olkin (KMO) and Bartlett's Test.

KMO	0.933	
Bartlett's Test	Approx. Chi-square	6587.56
	df	780
	Sig	0.000

**Table 4:** The results of performing exploratory factor analysis test on the Burnout Factors in Operating Room Nurses.

Item Number	Factor Load	The title of the item
Factor 1: organizational factors		
Item 3	0.686	When the decision in the operating room is made by non-specialists, it is unbearable for me
Item 5	0.769	I am under pressure because the organization does not support me enough in the work environment
Item 6	0.725	Lack of job promotion makes me feel unmotivated
Item 8	0.580	If there are no proper instruments and equipment in the operating room, I bear more work pressure
Item 9	0.572	I get annoyed if there are no standard environmental conditions (such as air conditioning, lighting, temperature) in the operating room
Item 10	0.501	The disproportion between salary and work duties makes me demotivated
Item 11	0.538	Unrelated tasks in the operating room make me tired
Item 12	0.505	Due to the insufficient number of nurses, I bear more pressure
Item 13	0.412	I get annoyed if the work schedule of the operating room is irregular
Item 18	0.427	In case of insufficient skill of the surgeon, I tolerate more pressure
Item 19	0.415	If there is no empathy between operating room nurses, I get annoyed
Item 21	0.387	If my colleagues are insufficiently skilled in their duties, I get more tired
Factor 2: individual factors		
Item 27	0.690	It is hard for me to bear working in the closed space of the operating room and not being able to move between departments
Item 28	0.632	I get upset when I see people's misconceptions about my job
Item 30	0.611	I feel inefficacity because I don't imagine a better work future for myself
Item 31	0.383	When a patient dies in the operating room, I endure severe mental and emotional pressure
Item 32	0.703	I get tired when I do repetitive activities in the operating room
Item 33	0.477	I suffer from not being able to show my true feelings in the operating room
Item 34	0.590	When I compare my conditions (economic, social, etc.) with the surgeon, I get disappointed
Item 35	0.356	I get annoyed when have to keep silent when I see the inappropriate behavior of managers
Item 36	0.504	Due to economic problems, I have to work more
Factor 3: interpersonal factors		
Item 14	0.381	If I don't pay my salary on time, I will be pressured
Item 15	0.645	I bear a lot of pressure in the operating room if the surgeon become angry
Item 16	0.900	When the self-esteem and personality of the nurses are ignored by the surgeon, I get annoyed
Item 17	0.708	Due to the bullying and dominance of the surgeon in the operating room, I bear more work pressure
Item 20	0.314	The presence of hypocrisy behavior in colleagues is painful for me
Factor 4: occupational nature factors		
Item 23	0.773	In taking care of patients with emergency conditions, I tolerate more mental and emotional pressure
Item 24	0.758	The possibility of physical and mental problems due to the stress in the operating room worries me
Item 25	0.598	Unexpected surgical events (such as lost sponges or surgical instruments) cause more work pressure on me
Item 26	0.334	I bear a lot of pressure while working at night shift
Item 38	0.480	It is difficult for me to move heavy instruments and equipment
Factor 5: managerial factors		
Item 1	0.842	The behavior of head nurse in the operating room with surgeons and nurses is discriminatory
Item 2	0.829	Lack of attention to my physical and mental conditions by head nurse is painful for me
Item 4	0.810	If I criticize of the head nurse, more pressure will be imposed on me

### Reliability

Cronbach's alpha coefficient of the whole 34-item questionnaire was equal to 0.937. In Table 5, Cronbach's alpha of all dimensions is shown. Therefore, all dimensions have a suitable reliability coefficient. Also, the ICC of the questionnaire was obtained with test–retest, 0.946.

**Table 5:** Reliability coefficient of the five dimensions of the burnout factors questionnaire in operating room nurses.

No	Dimensions	Item	Cronbach's alpha
1	Burnout related to organizational factors dimension	12(3-5-6-8-9-10-11-12-13-18-19–21)	0/87
2	Burnout related to individual factors dimension	9(27-28-30-31-32-33-34-35-36)	0/84
3	Burnout related to interpersonal factors dimension	5(14-15-16-17-20)	0/78
4	Burnout related to occupational nature factors dimension	5(23-24-25-26-38)	0/80
5	Burnout related to managerial factors dimension	3(1-2-4)	0/84

## Discussion

Burnout questionnaire of operating room nurses was designed based on the concept expressed with 34 items and in 5 dimensions. In this study, face validity (qualitative and quantitative), content validity (qualitative and quantitative), construct validity (factor analysis), internal consistency and stability of this instrument were confirmed.

In the present study, 65 items were initially designed using interviews and literature review. In the phase of determining qualitative face validity, the comments of operating room nurses were applied and four items were edited in terms of writing. Also, in the quantitative section, all the items had Item impact higher than 1.5, so all of them were retained. According to this step, the designed questionnaire has good face validity. Norful et al.^43^, Kristensen et al.^44^ and Mahmoudi et al.^45^ also used face validity to check the validity of the job burnout questionnaire.

To survey the content validity of the questionnaire, three parts were used, including the qualitative method, Content Validity Ratio (CVR) and Content Validity Index (CVI). At the end of the qualitative phase, 14 items were edited, 3 items were removed, 5 items were merged and one item was added. In the CVR section, 20 items were removed, and finally, all the items were retained by checking the CVI. Also, Scale-CVI/Ave of the questionnaire was calculated, which had an acceptable score (0.97). Polit et al. have recommended a score of 0.90 and above to accept Scale-CVI/Ave^33^. Mahmoudi et al.^45^, Salaree et al.^25^ and Sharifi et al.^46^ used CVR and CVI to check the content validity of the job burnout questionnaire and reported the results as appropriate.

The construct validity of the burnout questionnaire was checked with exploratory factor analysis on the remaining 40 items. The results of the KMO index and Bartlett's Test confirmed the factor analysis model and finally, according to the Eigenvalue, 5 factors were extracted.

In the present study, Cronbach's alpha coefficient of 0.937 along with test–retest showed the appropriate reliability of the designed tools. In line with the present study, Consiglio et al.^47^, Javanshir et al.^48^ and Salaree et al.^25^ investigated the construct validity and reliability of the burnout questionnaire using exploratory factor analysis and Cronbach's alpha coefficient which reported the results as acceptable.

In this study, the concept of burnout was investigated and explained from the perspective of the participants. Previous studies have shown that burnout is a mental concept and because the mind is affected by the body, so if the body is stressed for any reason like overwork, the mind also gets tired. Therefore, researchers have emphasized that when investigating burnout, checking the work environment is also very important because a high stress work environment indicates an increase in the level of burnout in personnel^49,50^. The present study showed that the concept of burnout in operating room nurses is a subjective concept that is influenced by Organizational, Individual, Interpersonal, Occupational Nature and Managerial factors, so that these factors play an important role as factors that cause burnout in operating room nurses. Factors name were based on the content of the items and experts' comments (nursing professor, surgeon, psychologists and expert in psychometrics of the questionnaire).

In the current study, organizational factors of job burnout had the highest factor load. The operating room is one of the most stressful environments and even a small mistake can cause serious harm to the patient. Therefore, in such a situation, if proper support is not provided by the organization, the nurses in the operating room bear a lot of pressure and may go towards burnout. In line with the present study, in previous studies, the important role of organizational factors such as lack of organizational support, organizational injustice, and inappropriate organizational performance has been emphasized in creating burnout of nurses^50,51^. In the dimension of individual factors, things such as job dissatisfaction, lack of motivation and tolerance of more work due to economic concerns may lead to increased burnout of operating room nurses. In line with the present study, the role of these factors in nurses' burnout has been mentioned in the research conducted by Bakaç et al.^52^ and Guo et al.^53^.

According to the results of the present study, operating room nurses may experience burnout through other factors such as interpersonal factors. In line with the results of the present study, due to the importance of communication between surgeons and nurses in the stressful environment of the operating room^54^ and teamwork in surgery^55^, if this dimension is not paid attention to, it may cause burnout of nurses. Also, the role of factors related to Occupational Nature and Managerial factors in creating burnout of operating room nurses should not be ignored. In line with the results of the present study, high work stress in the operating room^17^, occupational hazards in this environment^56^ and inappropriate performance of managers^57^ can cause burnout of nurses.

One of the most widely used tools to measure burnout in internal and external studies is the Maslach Burnout Inventory, but this tools is designed for general use and has low sensitivity for use in specific groups^25,58^. Therefore, in order to measure job burnout more accurately, it seems necessary to design and use specific tools.

One of the strong points of this study is the design of a specific tools for burnout factors of operating room nurses based on the concept of burnout in the mentioned society and its validity and reliability. The limitation of this study is sampling in Iran, and due to the social, cultural and geographical factors in people's experiences that were effective in the design of the tool, its generalization to other societies should be done with caution. Also, one of the other limitations of the present study is the lack of use of EFA in construct validity. In addition, the self-reporting of the participants about lack of a history of previous psychological diseases can be one of the limitations of the present study.

## Conclusions

Based on the results obtained from this study, a questionnaire of job burnout factors in operating room nurses was designed with 34 items. This tool was designed using two qualitative and quantitative approaches, i.e., interview and comments of nurses and experts in different fields along with the use of different validation methods, and it has good validity and reliability. For future studies considering that the present questionnaire can be easily used to measure nurses' burnout, it is recommended researchers using the present questionnaire to measure burnout and also conducting an intervention study such as designing educational programs and necessary policies to reduce it in operating room nurses.

## Data Availability

The datasets used and/or analysed during the current study are available from the corresponding author upon reasonable request.

## Abbreviations

CVR: Content validity ratio
CVI: Content validity index
KMO: Kaiser–Meyer–Olkin
EFA: Exploratory factor analysis
PCA: Principal component analysis
ICC: Intraclass correlation coefficient
